# A survey on spinal cord injuries resulting from stabbings; a case series study of 12 years’ experience

**DOI:** 10.5249/jivr.v5i1.277

**Published:** 2013-01

**Authors:** Hamid Reza Saeidiborojeni, Mehdi Moradinazar, Sepehr Saeidiborojeni, Alireza Ahmadi

**Affiliations:** ^a^Imam Reza Hospital, Kermanshah University of Medical Sciences, Kermanshah, Iran.; ^*b*^Imam Khomaini Hospital, Kermanshah University of Medical Sciences, Kermanshah, Iran.; ^*c*^Tehran University of Medical Sciences, Tehran, Iran.; ^*d*^Kermanshah University of Medical Sciences, Department of Anesthesiology, Critical Care and Pain Management, Kermanshah, Iran.; ^*e*^Department of Public Health Sciences, Division of Social Medicine, Karolinska Institute, Stockholm, Sweden.

**Keywords:** Spinal cord injury, Stab wound, Penetrating

## Abstract

**Background:**

Penetrating spinal cord injuries (SCIs) are an uncommon injury and not reported very frequently. SCIs cause sensory, motor and genitourinary system problems or a combination of sensorimotor dysfunctions. These are among the most debilitating kinds of disorders and negatively affect quality of life, not only for the patient, but also for their family members. Therefore, the present study aims to evaluate complete or incomplete SCIs and the course of the injury and the prognosis for SCIs caused by stab wounds.

**Methods:**

This case-series design study was performed on 57 patients attending the emergency department of Taleqani Trauma Center (Kermanshah, Iran) due to SCIs caused by violent encounters involving sharp objects such as a knife, dagger, whittle and Bowie-knife between 1999 and 2011. An assessment of sensory and motor functions was performed as part of the neurological examination on admission, and during the treatment, using the Frankel Classification grading system, and the results were recorded.

**Results:**

The average age of patients was 27 years (SD= 7.9, Range=17 to 46 years). The results of the study showed a proportion of cervical, thoracic and lumbar injuries of 23 (40%), 24 (42%) and 10 (18%), respectively. There was no case of cerebrospinal fluid leakage (CSF) or infection at the wound site in the subjects. Regarding the extent of the SCI, the combined neurological assessment showed that several patients (43%) had a complete SCI with no sensory and motor functions in the sacral segments and the segments below the site of injury. In 32 patients (57%) incomplete injuries were observed; i.e. they showed only some degrees of sensory-motor functions that were below the neurological level.

**Conclusions:**

Both complete and incomplete SCIs are of great importance because the prognosis of SCI is directly associated with the location and extent of injury. It should be considered that partial recovery from SCIs is possible in few cases of complete injuries. Therefore, all the patients should be treated carefully and seriously.

## Introduction

Penetrating spinal cord injuries (SCIs) are an uncommon medical problem and not reported as frequently as SCIs caused by motor vehicle accidents and falls.^1^ SCIs cause sensory, motor and genitourinary system problems or a combination of sensorimotor dysfunctions. These injuries are among the most debilitating conditions and negatively affect quality of life, not only for the patient but also for their family members.^2^ SCIs are observed mostly in young people and have an important role in diminishing life expectancy.^3,4^ World Health Organization (WHO) defines SCI as any damage to the spinal cord that results in a partial or complete impairment of the spinal performance including sensory, motor, autonomic and reflex functions completely or incompletely.^5^ Therefore, considering the importance of the issue and the lack of comprehensive studies on penetrative spinal injuries, we aimed to evaluate SCIs and the course of the injury and the prognosis for SCIs caused by sharp objects during violent encounters.

## Methods

**Participants**

**Figure 1:Cervical spinal cord injury due to stabbing.**
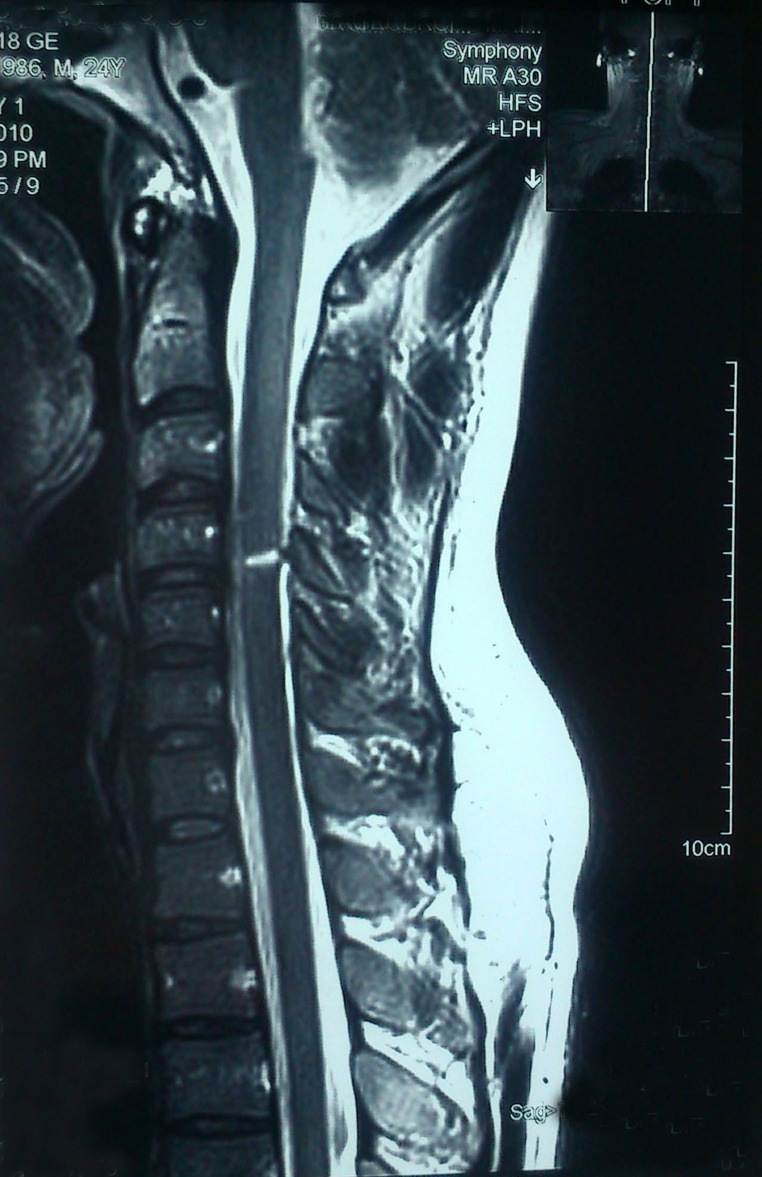


**Figure 2: Thoracic spinal cord injury by penetrating metallic knife.**
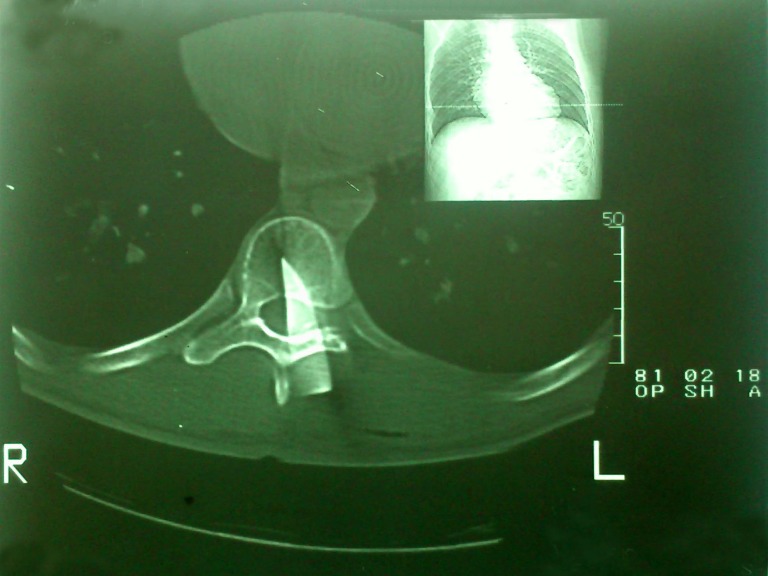


The study was performed on 57 patients with SCIs admitted to the emergency department of Taleqani Hospital (Kermanshah, Iran) because of the SCIs sustained from stabbing incidents with sharp objects such as knifes, daggers, whittle and Bowie knives between 1999 and 2011. In 56 patients, the sharp object was removed from the body before arrival at the hospital. Complete and systematic examination was performed in terms of vascular, abdomen and pulmonary injuries and simple radiography of the spine was started. After an improvement of the general condition of the patient, spinal MRI was done on the first day of admission and then during the treatment if no improvement was seen in order to rule out other complications (Figure 1). In one of the patients, because of the existence of an external, metallic object in the spine, a CT scan of the spine was performed (Figure 2). All patients underwent surgical debridement and repairing of anatomical covering layers. Although the controversy persists about the use of a high dose of methylprednisolone infusion for acute SCI, we prescribed methylprednisolone and antibiotics and analgesic for patients.^6,7^ The method of administration was a bolus intravenous infusion of 30 mg per kg of body weight over fifteen minutes within eight hours of SCI, followed 45 minutes later by an infusion of 5.4 mg per kg of body weight per hour for 23 hours. There is insufficient evidence to support the continuation of this treatment beyond 23 hours.^8,9^

**Protocol**

The current study was a case series design and, a standard neurological assessment of sensory and motor functions was performed initially and during follow-up. Motor functions were evaluated by testing of each muscle and coring the results from zero to 5 and multiple sensory examinations were performed for evaluation of the spinothalamic tract (pain and temperature senses) and the posterior tract (touch, vibration and condition). In this study, for classification of the SCI, the Frankel grading method was applied concurrently. The patients were divided into two groups:

1. Complete lesions: There was no function (sensory or motor) below the level of the lesion. Complete injury is mostly related to spinal transection. In complete lesions there are losses of sensory and motor function due to the spinal transaction from the brain.

2. Incomplete lesions: There was some functioning below the primary level of the injury. An injury was defined as neurologically incomplete if there was any sparing present at the lowest sacral segments.^10^ This was done by examining rectal and touching sacral segment. If voluntary contraction of anal sphincter is observed, it is called incomplete lesion. The patients attending the study were assessed monthly until one year after injury.

## Results

**Figure 3: Final results of 57 stab wounds of spinal cord patients admitted to Kermanshah Trauma Center 1999 -2011:**
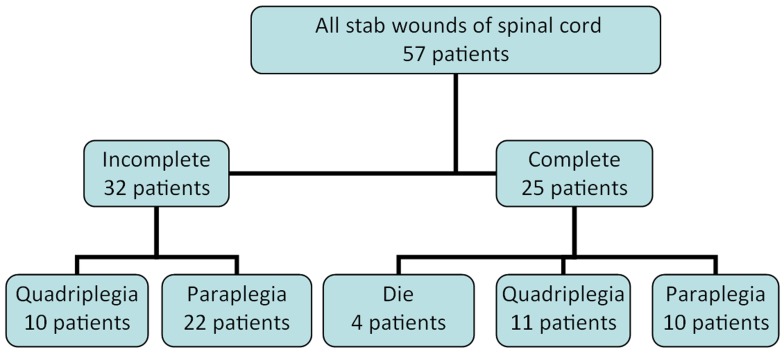


The average age of patients was 27 years (SD= 7.9, Range=17 to 46 years). There were 48 men (82%) and 9 women (12%). 44 persons (75%) were single and 13 persons (25%) were married. 46 patients (80%) had an educational level lower than high school graduate. 5 patients (8%) had completed pre-university studies and only 6 patients (10%) had an academic education. 22 patients were unemployed, 23 workers, 8 homemakers and 4 patients were students of university or school (Figure 3).

The results of the study showed that the proportion of cervical, thoracic and lumbar injuries was 23 (40%), 24 (42%) and 10 (18%), respectively. There was no case of cerebrospinal fluid leakage (CSF) or infection at the wound site in the subjects. As is shown in Figure 3, regarding the extent of the SCI, the combined neurological assessment showed that 25 patients (43%) had a complete SCI and 32 patients (57%) had an incomplete SCI. Of the 25 patients with complete injuries, 4 patients (16%) died because of respiratory problems, not having responded to the use of the artificial respiratory device and medicinal therapy. In 10 patients (40%), paresis improved resulting in approximately 6 patients in grade C of the Frankel scale and 4 patients in grade D of the Frankel scale. These patients could more or less continue their normal daily activities with some help. However they had urinary- fecal incontinence and sexual problems. In 11 (54%) patients with complete SCI, not responding to the frequent medicinal and physiotherapy treatments, no improvement of neurological condition was observed. Paralysis and fecal/urinary incontinence and sexual problems of the patients continued. In 32 patients with incomplete injury, the recovery process was better, particularly in patients with Brown-Sequard Syndrome (BSS) such that 22 out of these 32 patients (69%) showed a relatively complete recovery and returned to their normal lives. The other 10 patients (31%) had incomplete recovery with some degrees of paresis. Their motor power was 3.5 but there was no fecal/urinary incontinence.

## Discussion

The epidemiological characteristics of the patients with SCI in the study were mostly similar to the studies performed in other parts of the world. In this study, most of the patients were men, single and unemployed, therefore the results were in line with the studies conducted in South Africa by Peacock et al. The mean age of the participants of our study was lower than the previous similar studies; this difference can be ascribed to the rather young population of Iran.^1,11^ In this study, most of the SCIs were incomplete and the results, particularly in patients with BSS, were in line with the previous studies.^11,12^ Approximately 7% of the patients with SCIs died because of a respiratory system dysfunction, the mortality rate reported in similar studies was approximately 4%. However, about 44% of the patients with complete SCIs recovered; it was the highest improvement rate compared to similar studies, although in 26% the recovery was not significant.^1,13^ Regarding the incomplete SCIs, our study demonstrated that approximately 70% of the patients improved, indicating a good consistency with the previous studies with the improvement ranging between 50% and 70%.^11,14,15^ The results of our study showed that most of the SCI sites were located in the lower part of the spine compared to the previous studies. In the similar studies, the injury was cervical (54-63%), thoracic (27-30%) and lumbar (< 7%). The proportion of cervical, thoracic and lumbar injuries was 40%, 42% and 18%, respectively. This is of great importance because the complications increase with injuries to upper parts of spine.^5,16^

There was no case of cerebrospinal fluid leakage (CSF) or infection at the wound site in the subjects. However, in a comprehensive study conducted in South Africa, about 4% of the patients experienced CSF leakage. In this case, the findings of our study were not consistent with this study.^1,11^ One of the important findings of our study was the importance of simple radiography of the spine in order to reveal any fragments of sharp objects in the body of the patient even though there was no sign on the skin. Therefore, a Computed Tomography (CT) scan was selected instead of Magnetic Resonance Imaging (MRI) method as the first choice of imaging modality in SCIs, in accordance with similar studies, It should however be considered that with the presence of external, metallic objects within the body, MRI can cause a strong deficiency of neural cells.^5^ The results of the study showed that the complete or incomplete injuries are of importance because the prognosis of injuries is directly associated with the extent of lesion. The prognosis of incomplete injury is usually easier to predict. One should remember that complete injury can improve. But the prognosis of patients with complete SCI is unpredictable.^17,18^ Completeness or incompleteness of the injury didn’t have any effect on the treatment outcome.^3^ All the patients with SCIs were brought to hospital by friends and acquaintances. If the patients had received first aid and had been immobilized before admittance, the sequelae were reduced. An important point is that, according to our questionnaire, before the accident 50 of the 57 victims (88%) were not aware of the nature and serious complications of SCI. Public Health Campaign aimed at informing the public, particularly young people and their families, about the SCIs’ potential complications to prevent these problems is important.
